# Proline metabolism reprogramming of trained macrophages induced by early respiratory infection combined with allergen sensitization contributes to development of allergic asthma in childhood of mice

**DOI:** 10.3389/fimmu.2022.977235

**Published:** 2022-09-20

**Authors:** Hanglin Li, Linyan Ma, Wenjian Li, Boyang Zheng, Junhai Wang, Shunyan Chen, Yang Wang, Fei Ge, Beibei Qin, Xiaoqing Zheng, Yuqing Deng, Ruihong Zeng

**Affiliations:** ^1^ Department of Immunology, Hebei Medical University, Shijiazhuang, China; ^2^ Key Laboratory of Immune Mechanism and Intervention on Serious Disease in Hebei Province, Hebei Medical University, Shijiazhuang, China; ^3^ The Fourth Affiliated Hospital of Hebei Medical University, Shijiazhuang, China; ^4^ Clinical Lab, Hebei Provincial People’s Hospital, Shijiazhuang, China

**Keywords:** allergic asthma, trained macrophages, innate immune memory, proline metabolism reprogramming, respiratory virus infection

## Abstract

**Background:**

Infants with respiratory syncytial virus (RSV)-associated bronchiolitis are at increased risk of childhood asthma. Recent studies demonstrated that certain infections induce innate immune memory (also termed trained immunity), especially in macrophages, to respond more strongly to future stimuli with broad specificity, involving in human inflammatory diseases. Metabolic reprogramming increases the capacity of the innate immune cells to respond to a secondary stimulation, is a crucial step for the induction of trained immunity. We hypothesize that specific metabolic reprogramming of lung trained macrophages induced by neonatal respiratory infection is crucial for childhood allergic asthma.

**Objective:**

To address the role of metabolic reprogramming in lung trained macrophages induced by respiratory virus infection in allergic asthma.

**Methods:**

Neonatal mice were infected and sensitized by the natural rodent pathogen Pneumonia virus of mice (PVM), a mouse equivalent strain of human RSV, combined with ovalbumin (OVA). Lung CD11b^+^ macrophages in the memory phase were re-stimulated to investigate trained immunity and metabonomics. Adoptive transfer, metabolic inhibitor and restore experiments were used to explore the role of specific metabolic reprogramming in childhood allergic asthma.

**Results:**

PVM infection combined with OVA sensitization in neonatal mice resulted in non-Th2 (Th1/Th17) type allergic asthma following OVA challenge in childhood of mice. Lung CD11b^+^ macrophages in the memory phage increased, and showed enhanced inflammatory responses following re-stimulation, suggesting trained macrophages. Adoptive transfer of the trained macrophages mediated the allergic asthma in childhood. The trained macrophages showed metabolic reprogramming after re-stimulation. Notably, proline biosynthesis remarkably increased. Inhibition of proline biosynthesis suppressed the development of the trained macrophages as well as the Th1/Th17 type allergic asthma, while supplement of proline recovered the trained macrophages as well as the allergic asthma.

**Conclusion:**

Proline metabolism reprogramming of trained macrophages induced by early respiratory infection combined with allergen sensitization contributes to development of allergic asthma in childhood. Proline metabolism could be a well target for prevention of allergic asthma in childhood.

## Introduction

Asthma is a major public health problem that affects 300 million people worldwide, among which are a large proportion of children (1). Aeroallergen sensitization is one of the strongest asthma risk factors, and most frequently acts in synergy with other proinflammatory environmental cofactors, most notably respiratory viral infections, to drive disease development (2). The most severe childhood asthma, and the type that confers the highest ensuing risk for progression to persistent asthma, is encountered when lower respiratory infections occur against a background of pre-existing aeroallergen sensitization, in particular during the period when postnatal lung growth and differentiation are proceeding most rapidly (2, 3). Increasing evidence is emerging to suggest that viral respiratory infections in early life are related with the medium and long-term development of asthma (4, 5). RSV is the most important respiratory pathogen for infants. RSV infects more than 60% of children in their first year of life (6). Epidemiological studies have reproducibly implicated RSV-bronchiolitis as a major risk factor for the onset and progression of asthma (7). Infants hospitalized with RSV-associated bronchiolitis, or even to mild RSV disease are at increased risk of asthma later in childhood (8).

The association between bronchiolitis caused by RSV and the development of recurrent wheezing and/or asthma was first described more than 40 years ago, but the underlying mechanisms are unclear. Elucidating the underlying mechanisms of the childhood asthma associated with respiratory virus infection in early life is critical for developing effective asthma treatments. A growing concern is that viral infections during the neonatal period perturb immunological homeostasis in the airways, and is responsible for an immunopathological memory in the lungs that could influence the severity of asthma in childhood. In newborns, lung myeloid cells are the key cell populations for immune tolerance and immune response (9). At postnatal day 7, myeloid cells, such as monocytes and macrophages reach adult-like cell frequencies, while lymphoid cells have not reached half of the maximum (9). A persistent post viral effects on the population dynamics of lung myeloid cell populations and their functional phenotype have been confirmed in parainfluenza and RSV infected mouse models (10, 11). Recent studies demonstrated that certain infections and vaccinations induce innate immune memory (also termed trained immunity), especially in macrophages, to respond more strongly to future stimuli with broad specificity (12), resulting in non-specific protection against reinfection (13), or involving in human inflammatory diseases (14). Metabolic reprogramming, such as glucose metabolism and amino acid metabolism, which increases the capacity of the innate immune cells to respond to a secondary stimulation, is a crucial step for the induction of trained immunity (15). The characterization of the differential metabolic responses of the different cells depends on the type of stimulus, cell subset, or tissue microenvironment (15). We hypothesize that specific metabolic reprogramming of lung trained macrophages induced by neonatal respiratory virus infection contributes childhood allergic asthma, which may provide a prevention or treatment target for allergic asthma in childhood.

In this study, the natural rodent pathogen Pneumonia virus of mice (PVM), a mouse equivalent strain of human RSV, was used to infect neonatal mice (16), followed with allergen ovalbumin (OVA) sensitization as a co-factor. The mice showed non-Th2 (Th1/Th17) type allergic asthma following OVA challenge. PVM infection combined with OVA sensitization (PVM-OVA) induced lung trained macrophages, which mediated the allergic asthma in childhood. Notably, proline metabolic reprogramming is crucial for development of the trained macrophages as well as the allergic asthma. Inhibition of excessive proline synthesis prevented childhood allergic asthma.

## Materials and methods

### Mice

Specific pathogen-free BALB/c suckling mice (5 days post birth) and female mice (4 or 6 weeks) were obtained from Hebei Laboratory Animal Center (Shijiazhuang, China), housed and manipulated according to the Care and Use of Laboratory Animals (China). All animal experiments were approved by the “Laboratory Animal Ethical and Welfare Committee Hebei Medical University” under number IACUC-Hebmu-2020010.

### Virus

PVM stain 15 was obtained from the American Type Culture Collection (ATCC), passaged in Six-week-old BALB/c mice, and titrated in BHK21 cells. The titer was determined by reed-Muench method.

### Animal treatment

Mice were treated as **Table 1**. Suckling mice were sensitized by PVM (2500 TCID50) and/or OVA (0.02%, 5 µl per mouse, In vivoGen, USA), or along with 10 μ g/g BPTES (HY 12683, MCE, NJ, USA) or 50 μg/g NFLP (N-Formyl-L-proline, Department of Medicinal Chemistry, Hebei Medical University), and 0.2mg/g Proline (Energy Chemical, Shanghai, China). Mice were sacrificed for testing immune memory on day 20, or challenged daily from day 21 to 25 with OVA (0.4%,50ul) intranasally for testing airway hyperreactivity and lung inflammation.

**Table 1:** Animal sensitization and challenge protocol.

	Infection(5d post birth)0d	Sensitization3d	Sensitization4d	Challenge21-25d
PBS	PBS i.n.	PBS i.n.	PBS i.n.	OVA i.n.
PBS-OVA	PBS i.n.	OVA i.n.	OVA i.n.	OVA i.n.
PVM	PVM i.n.			OVA i.n.
PVM-OVA	PVM i.n.	OVA i.n.	OVA i.n.	OVA i.n.
PVM-OVA+BPTES	PVM i.n. + BPTES i.p.	OVA i.n. + BPTES i.p.	OVA i.n.	OVA i.n.
PVM-OVA+NFLP	PVM i.n. + NFLP i.p.	OVA i.n.+ NFLP i.p.	OVA i.n.	OVA i.n.
PVM-OVA + BPTES + Pro	PVM i.n. + BPTES i.p. + Pro i.p.	OVA i.n. + BPTES i.p. + Pro i.p.	OVA i.n.	OVA i.n.
PVM-OVA + NFLP+ Pro	PVM i.n. + NFLP i.p. + Pro i.p.	OVA i.n. + NFLP i.p. +Pro i.p.	OVA i.n.	OVA i.n.

### Airway hyperreactivity

Lung resistance index (RI) and dynamic compliance (Cdyn) in response to increasing doses of inhaled methacholine (Mch, doses: 6.25,12.5,25, and 50 mg/ml) was assessed using Buxco FinePointe Mouse RC site. Data are expressed as a percentage change from baseline.

### BALF collection

Bronchoalveolar lavages were performed as previously described (17). Briefly, bronchoalveolar lavage fluid (BALF) was collected by washing the lungs of a mouse with 700ul normal saline via bronchus. Collected BALF was centrifuged, the cell pellet was resuspended in PBS, and leukocytes were counted by standard optical microscope or cell types were analyzed by flow cytometry.

### Flow cytometry

Lung single cells were incubated with fluorescein labeled antibodies, anti-mouse CD11b-PE, CD69-FITC, TLR4-APC, CD49a-PE-vio770, Gr-1-FITC (MACS, Miltenyi, Germany). The cells were analyzed on flow cytometry (BD), and FACS data were analyzed using Cell Quest software (Becton Dickinson).

### Histopathology

Lung tissues were fixed in 4% paraformaldehyde. Paraffin-embedded sections were stained using Hematoxylin & Eosin (H&E) or Periodic Acid Schiff (PAS), then scored blindly as previously described (18, 19). The lungs then were embedded in paraffin, sectioned, and stained with hematoxylin/eosin or periodic-acid Schiff reaction mixture for detection of mucin. The severity of inflammation was evaluated separately for the alveolar and peribronchial tissue and perivascular spaces in a group-blind fashion. The degree of inflammation in the alveolar tissue was graded as follows: 0, normal; 1, increased thickness of the interalveolar septa (IAS) by edema and cell infiltration; 2, increased thickness of IAS with presence of luminal cell infiltration; 3, abundant luminal cell infiltration; and 4, inflammatory patches formed. The degrees of inflammation in the peribronchial and perivascular spaces were graded as follows: 0, no infiltrate; 1, slight cell infiltration noted; 2, moderate cell infiltration noted; and 3, abundant cell infiltration noted. The mucus secretion was assessed on PAS-stained sections and consists in general of mucopolysaccharides staining at the apical pole of goblets cells in the peribronchial area, which was graded using a semi-quantitative score from 0 to 5 with increasing grade of infiltration by 2 observers independently as follows: 1 = Normal lung; no mucus; 2 = Mucus in 1/3 of the bronchi; 3 = Mucus in 1/2 of the bronchi; 3 = Mucus in 2/3 of the bronchi; 4 = Mucus in 3/4 of the bronchi; and 5 = Mucus everywhere in the bronchi.

### Cell isolation

Lung tissue was digested with collagenase type IV, and lung single cells were obtained by grinding on 200-mesh copper mesh.CD11b+ macrophages were sorted using Magnetic Bead Sorting Kit (MACS, Miltenyi, Germany).

### Adoptive transfer and challenge

Isolated CD11b+ macrophages were washed and resuspended with 200 μl of PBS, and injected into a 4-week-old BALB/c mouse via the tail vein (5×10^5^ cells per mouse). After 24 h, mice were challenged i.n. with 50 μl of 0.4% OVA for 5 consecutive days.

### Real-time RT-PCR

Total RNA was isolated using TRIZOL. Reverse transcription was performed with Super Script III Kit (Thermo Scientific, U.S.A). Cytokines were assessed by real-time PCR with SYBR green (Vazyme, U.S.A). The mouse housekeeping gene (β-actin) was used as a control. Primer sequences are listed in **Table 2**.

**Table 2:** Primer sequences form RNA analysis by real-time PCR.

β-actin	Forward	GCTACAGCTTCACCACCACAG
	Reverse	GCTCTTTACGGATGTCAACGTC
IL-1	Forward	TTCCTTGTGCAAGTGTCTGAAG
	Reverse	CACTGTCAAAAGGTGGCATTT
IL-5	Forward	CCCATGAGCACAGTGGTGAA
	Reverse	CTCATCGTCTCATTGCTTGTCAA
IL-13	Forward	CCTGGATTCCCTGACCAACA
	Reverse	GGGCCTTGCGGTTACAGA
IL-17	Forward	CCTCCAGAATGTGAAGGTCA
	Reverse	CTATCAGGGTCTTCATTGCG
TNF-α	Forward	TGACGTGGAACTGGCAGAAGA
	Reverse	TGGGCCATAGAACTGATGAGAG
IFN-γ	Forward	AGCAACAGCAAGGCGAAAAA
	Reverse	TGGTGGACCACTCGGATGA
MCP-1	Forward	GCTGACCCCAAGAAGGAATG
	Reverse	GAAGACCTTAGGGCAGATGCA
GRO-α	Forward	CATGTAGAAAGCCCATCTGGA
	Reverse	CTGCAATCAGAAAAGAGTCATTG
PYCR1	Forward	ACTCAGAACAGCATCCCAGC
	Reverse	TAAGCAAGGAGCGAAAGCCC
GLS1	Forward	GTCCTGAGGCAGTTCGGAATACAC
	Reverse	GAGGAGGAGACCAACACATCATGC
GLS2	Forward	GCCAGTTTGCCTTCCATGTG
	Reverse	GTCTAACTTCCGAGCGCAGT

### Metabolomics

The metabolites of the CD11b+ macrophages were identified by mass spectrometry (Shanghai Meiji Biology, China). Data were analyzed on the online platform of Majorbio Cloud Platform (www.majorbio.com). All the metabolites were compared with KEGG and HMDB databases. PLS-DA and OPLS-DA were used to capture the correlated differential variables, and then the differential metabolic sets obtained according to certain screening conditions (such as function, expression level and expression difference) were analyzed. Finally, the Major bio online tool was performed to explore biological patterns, functions, and pathways of identified differentially expressed metabolites.

### Statistical analysis

Measurement data were first tested for normal distribution using Kolmogorov-Smirnov methods. All data were normally distributed. Comparisons of data between two groups were analyzed by independent sample t test, and among multiple groups were analyzed by one-way analysis of variance (ANOVA). Data analyses were performed with GraphPad Prism 8 (GraphPad Software, La Jolla, CA, USA). P<0.05 is considered statistical significance.

## Results

### Neonatal PVM infection combined with OVA sensitization facilitated childhood asthma

**Figure 1:**
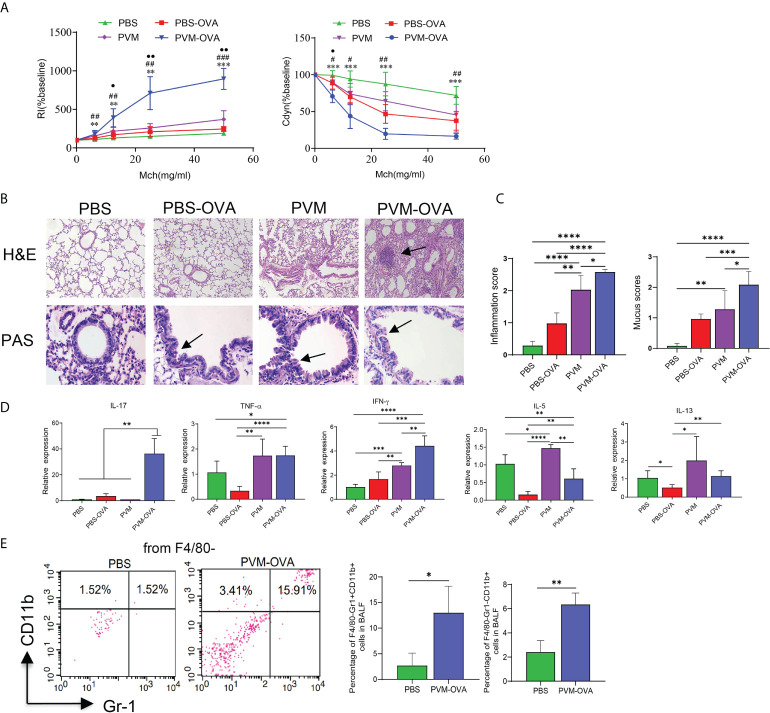
Neonatal PVM infection combined with OVA sensitization facilitated childhood allergic asthma in mice. **(A)** Airway resistance index (RI) and lung dynamic compliance (Cdyn) to increasing doses of inhaled methacholine (MCh, 6.25, 12.5, 25, and 50 mg/ml) following OVA challenge from day 21 to 25 post infection in mice. PVM-OVA group compared with PBS group, *, with PBS-OVA group, #, with PVM group, ●. **(B)** H&E staining shows peribronchiolar, perivascular, and interstitial inflammatory cell infiltration (100X). PAS staining shows bronchiolar mucus production (400X). **(C)** Scores for pulmonary inflammation and mucus. **(D)** Relative expression of cytokines in lung tissue by real-time PCR. **(E)** Expression of CD11b and/or Gr-1 on F4/80^—^ BALF cells in PBS and PVM-OVA group by flow cytometry. Each experimental group contained three biology repeats. Data are presented as means standard deviations of 5-6 mice per group and are representative of two experiments. Values are shown as means ± SEM. (*P<0.05, **P<0.01, ***P<0.001, ****P<0.0001 ^##^P<0.01, ^###^P<0.001, •P<0.05, ••P<0.01).

AHR is a prominent feature of asthma. Neonatal mice were sensitized and challenged as shown in **Table 1**. Following OVA challenge in childhood, mice early sensitized by PVM-OVA showed higher RI and lower Cdyn, compared with those by PBS or PBS-OVA (**Figure 1A**, P<0.01). RI in PVM group was higher than that in PBS group (**Figure 1A**, P<0.05), while no statistical difference on Cdyn between the two groups (**Figure 1A**, P>0.05). PVM-OVA group exhibited multiple severe peribronchial and perivascular inflammatory cell infiltration and mucus, while PVM group or PBS-OVA group exhibited moderate or slight inflammation and mucus (**Figure 1B**). Both inflammation and mucus scores in PVM-OVA group were higher than those in PVM, PBS-OVA, or PBS group (**Figure 1C**, P<0.05). The inflammation score in PVM group was also higher than that in PBS-OVA or PBS group. Both inflammation and mucus scores in PVM and PBS-OVA were higher than those in PBS group (**Figure 1C**, P<0.05). Relative expression of IL-17, TNF-α or IFN-γ was significantly higher in PVM-OVA group than that in PBS or PBS-OVA group (**Figure 1D**, P<0.05). However, expression of IL-5 was significantly lower in PVM-OVA group than that in PBS or PVM group (**Figure 1D**, P<0.05). No difference was observed in the expression of IL-13 between PVM-OVA group and PBS group (**Figure 1D**, P>0.05). Relative expression of IFN-γ or IL-5 was significantly higher in PVM group than that in PBS group (**Figure 1D**, P<0.05). No difference in all of these cytokine expressions was observed between PBS-OVA and PBS groups (**Figure 1D**, P>0.05). These data suggest that PVM infection in synergy with low dose OVA sensitization in early life led to severe asthma in childhood, which is similar to clinical childhood asthma (20, 21). In contrast, PVM infection alone only led to slight asthma, and OVA sensitization alone led to inflammatory histopathology, but no increased RI and cytokine expression. This finding is consistent with the results reported by R S Peebles Jr et al. (22).Th2-type cytokines were prevalent in eosinophilic asthma, whereas Th1-type and Th17-type cytokines have been implicated in neutrophilic asthma (23). In PVM-OVA group, Th1/Th17 type cytokines increased. So, we further tested the neutrophils and eosinophils in BALF. Neutrophils and eosinophils of PVM-OVA group were remarkably higher than those of PBS group, and notably, neutrophils of PVM-OVA group were about 6 times more than those in PBS group following OVA challenge (**Figure 1E**, P<0.05). There was no detectable IgE in sera (data no shown). The data suggested that early PVM-OVA sensitization resulted in asthma in childhood with Th1/Th17-biased responses and remarkably increased neutrophils.

### Lung CD11b+ trained macrophages increased after PVM-OVA early sensitization, and mediated asthma in childhood

**Figure 2:**
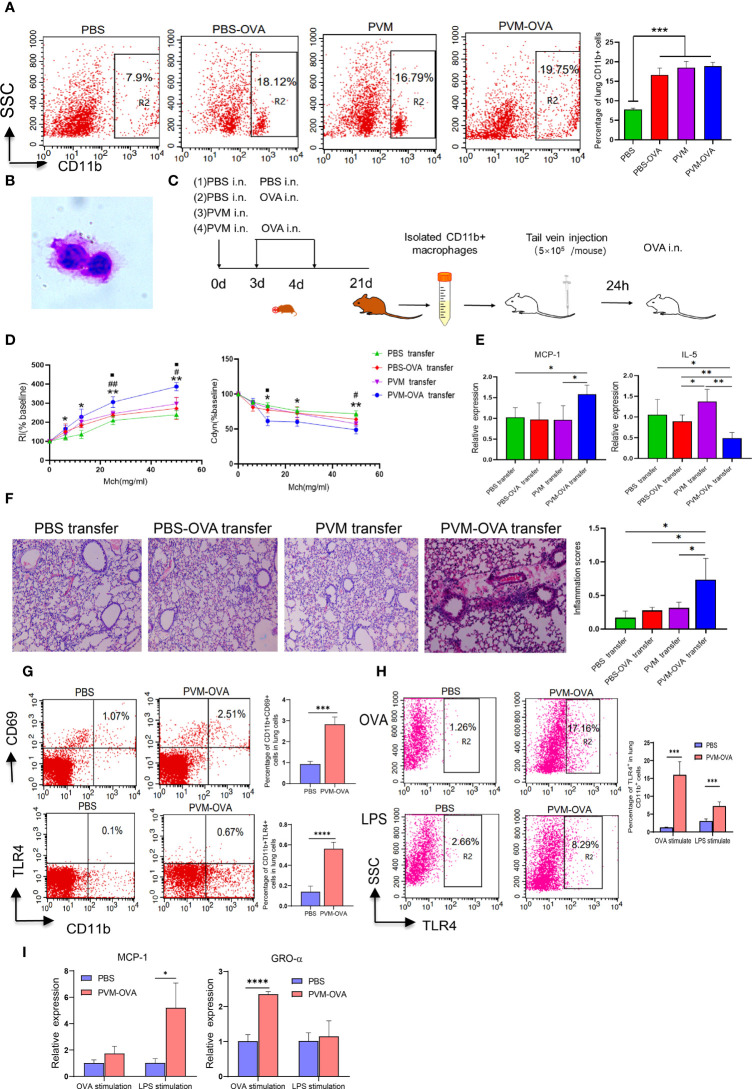
Lung CD11b^+^ trained macrophages increased after PVM-OVA early sensitization and mediated asthma in childhood mice. **(A)** The lung CD11b^+^ cells in the memory phase after sensitization by flow cytometry. **(B)** Lung CD11b^+^ macrophages morphologic evaluation. **(C)** Timeline of mouse adoptive transfer experiment. (1) PBS transfer group, (2) PBS-OVA transfer group, (3) PVM transfer Group, (4) PVM-OVA transfer group. **(D)** RI and lung Cdyn in mice after adoptive transfer of the lung CD11b^+^ macrophages following OVA challenge. PVM-OVA transfer group compared with PBS transfer group, *, with PBS-OVA transfer group, #, with PVM transfer group, <.**(E)** Relative expression of MCP-1 and IL-5 in lung tissue following OVA challenge by Real-time PCR. Each experimental group contained three biology repeats. Data are presented as means standard deviations of 5-6 mice per group. **(F)** H&E staining of lung tissue slices after adoptive transfer the lung CD11b^+^ macrophages (100X) and inflammation scores following OVA challenge. **(G)** CD11b^+^CD69^+^ macrophages or CD11b^+^TLR4^+^ macrophages in lungs by flow cytometry. **(H)** TLR4 expression of the sorted CD11b+ macrophages after LPS or OVA stimulation by flow cytometry. **(I)** Relative mRNA expression of chemokines MCP-1 or GRO-α of the sorted CD11b^+^ macrophages after LPS or OVA stimulation by real-time PCR. *P<0.05. ** P<0.01, ***P<0.001, **** P<0.001. ## means PVM-OVA transfer group compared with PBS-OVA transfer group (P<0.01). The symbol “■” but not “<” means PVM-OVA transfer group compared with PVM transfer group (P<0.05).

The data by flow cytometry showed that OVA or PVM alone induced significantly elevated CD11blow macrophages, while PVM-OVA induced more CD11bhigh macrophages, compared with PBS on day 21 (**Figure 2A**, P<0.05). Johnston LK et al. confirmed that CD11b high, CD11b int and CD11b low cells in lung tissue were all macrophages (24). CD11b on macrophages is the key regulatory factor of pro-inflammatory immune responses (25). Then, we sorted the CD11bhigh and CD11blow cells, performed cytospin, and confirmed that they were all macrophages in appearance (large, rounded nuclei with abundant cytoplasm) with a microscope, and representative images were shown (**Figure 2B**). To further assess a potential role in asthma, the macrophages were isolated and adoptive transferred to wild BALB/c mice of the same age (4 weeks post birth) by tail vein (**Figure 2C**). RI increased, and Cdyn decreased in PVM-OVA transfer group, compared with those in PBS transfer group following OVA challenge (**Figure 2D**, P<0.05). RI at 25 and 50mg/ml Mch was higher, and Cdyn at 12.5 or 50mg/ml Mch was lower in PVM-OVA transfer group, compared with those in PVM or PBS-OVA transfer group (**Figure 2D**, P<0.05). RI in PVM or PBS-OVA transfer group was same as that in PBS transfer group, except that at the doses of 12.5, and Cdyn in PVM or PBS-OVA transfer group was same as that in PBS transfer group at all doses of Mch (**Figure 2D**, P>0.05). MCP-1 expression was significantly higher, and IL-5 expression was inhibited in PVM-OVA transfer group, compared with those in PBS, PVM or PBS-OVA transfer group (**Figure 2E**, P<0.05), and there was no difference on relative expression of MCP-1 or IL-5 between PVM or PBS-OVA transfer group and PBS transfer group (**Figure 2E**, P>0.05). Histopathology showed that in PVM-OVA transfer group, the alveolar structure was destroyed, and the infiltration of inflammatory cells increased significantly, while the CD11blow macrophages from PVM or PBS-OVA group did not lead to inflammation histopathology (**Figure 2F**). These results suggest that the macrophages induced by PVM-OVA in early life mediated asthma in childhood, while the macrophages induced by PVM or OVA alone did not contribute to severe allergic asthma. This supports the conclusion that PVM infection in synergy with OVA sensitization in early life led to severe asthma in childhood, but not PVM infection or OVA sensitization alone (**Figure 1**). Therefore, we conducted all subsequent experiments using PVM-OVA but not PVM or PBS-OVA alone.

CD69 is a key marker of tissue resident memory T cells (26). We found that CD11b+CD69+ macrophages in PVM-OVA group were remarkable higher than those in PBS group (**Figure 2G**, P<0.001). A remarkable feature of trained immune cells is its ability to mount a stronger transcriptional response to nonspecific stimuli compared to untrained cells. The CD11b+ macrophages from PVM-OVA group expressed higher level of TLR4 after OVA or lipopolysaccharide (LPS) stimulation, compared with those from PBS group (**Figure 2H**, P<0.001). In contrast, the macrophages without in vitro stimulation in PVM-OVA group or PBS group expressed very low level of TLR4 (**Figure 2G**). Moreover, relative expression of MCP-1 in the macrophages from PVM-OVA group after LPS stimulation significantly increased, compared with that of PBS group (**Figure 2I**, P<0.05). Gro-α expression in PVM-OVA group after OVA stimulation significantly increased, compared with that of PBS group (**Figure 2I**, P<0.001). These results suggested that increased lung CD11b+ macrophages induced by PVM-OVA were trained macrophages.

### Lung trained macrophages undergo metabolic reprogramming

**Figure 3:**
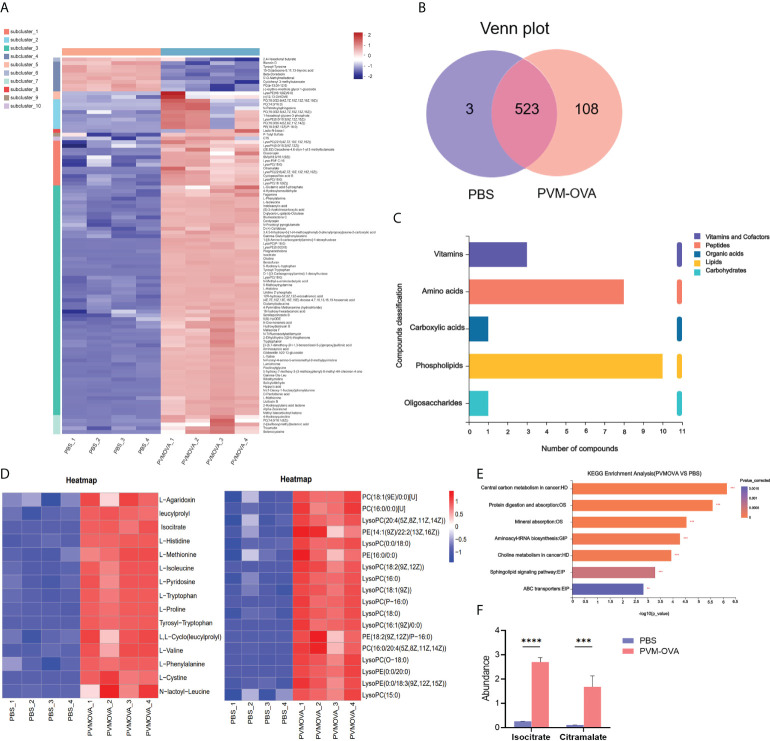
Lung CD11b^+^ trained macrophages undergo metabolic reprogramming. **(A)** Heat map of differential metabolites of PVM-OVA group vs PBS group. **(B)** Venn plot of the numbers of different metabolites among groups. The diagram shows overlapping and distinct metabolites indicated by the numbers in the intersections and circles, respectively. **(C)** KEGG compound classification of differential metabolites. **(D)** Heat maps of differential metabolite amino acids or phospholipids. **(E)** KEGG pathway enrichment analysis. **(F)** The abundance of metabolites related to TCA cycle. ***P<0.001, ****P<0.0001.

Trained immunity involves metabolic reprogramming, which endows innate immune cells with the ability to respond more strongly to a second stimulus (27). The CD11b+ macrophages in the memory phase were sorted and re-stimulated by LPS for metabolic analysis. As shown in **Figure 3A**, most of the metabolites markedly increased, and a few metabolites decreased in PVM-OVA group, compared with those in PBS group. There were 108 specific metabolites in PVM-OVA group, while there were 3 specific metabolites in PBS group, as found by Venn map/diagram analysis (**Figure 3B**). The KEGG compound classification analysis showed that these metabolites were grouped into biochemical classes including amino acids, phospholipids, vitamins, carboxylic acids and oligosaccharides (**Figure 3C**). Among the differential metabolites, the most abundant metabolite classes were amino acids and phospholipids (**Figure 3C**). Amino acids such as tryptophan, methionine, proline, and so on, and phospholipid metabolites including various phosphatidylcholine (PC) and phosphatidylethanolamine (PE) in PVM-OVA group were significantly increased, compared with those in PBS group (**Figure 3D**). Reprogramming of energy metabolism is the symbol of cancer, which meets the rapid growth of cancer cells (28). Metabolic pathway enrichment analysis in the KEGG database showed that the differential metabolites between PVM-OVA and PBS groups were most concentrated on central carbon metabolism in cancer (**Figure 3E**), which implied cancer cell-likely energy metabolism characteristic of the CD11b+ macrophages in PVM-OVA group. Consistently, quantity of isocitrate or citramalate in PVM-OVA group was markedly higher than that in PBS group (**Figure 3F**, P<0.05). These results suggested that the metabolism of the CD11b+ macrophages was reprogrammed, which further demonstrated the macrophages to be trained macrophages.

### Proline metabolism in mice sensitized with PVM-OVA was significantly reprogrammed

**Figure 4:**
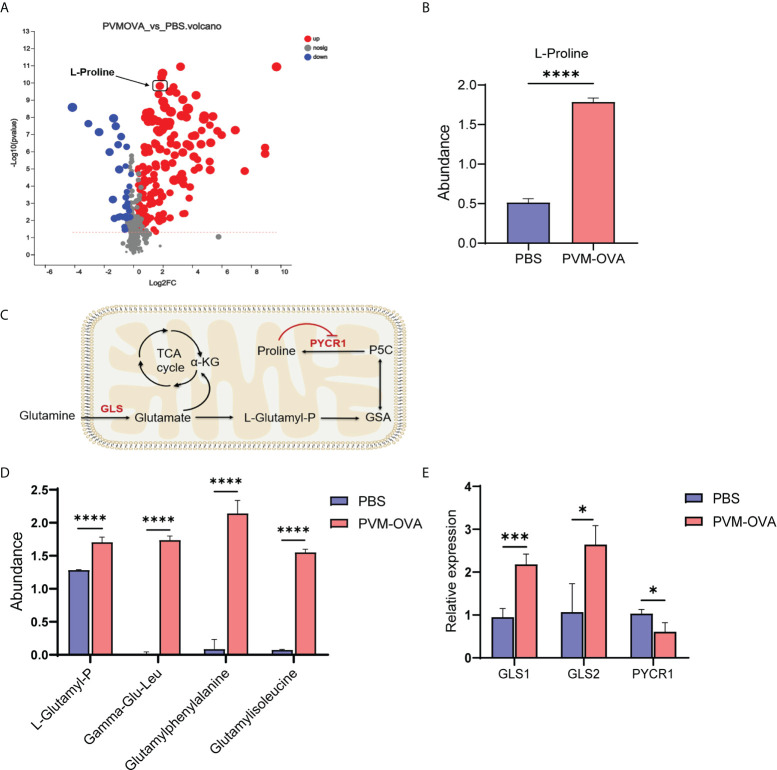
Proline metabolism in mice sensitized with PVM-OVA was significantly reprogrammed. **(A)** Volcanic map of differential metabolites of PVM-OVA group vs PBS group. **(B)** The abundance of proline. **(C)** Diagram of proline synthesis pathway from glutamine. **(D)** The abundance of dipeptide containing glutamic acid. **(E)** Relative expression of enzymes, GLS1, GLS2 and PYCR1, in the synthesis pathway of proline in the lung by real-time PCR. Each experimental group contained three biology repeats. *P<0.05, ***P<0.001, ****P<0.0001.

Evidences have shown that proline deficiency can significantly inhibit the growth of cancer cells (29, 30). Proline is a “limiting amino acid” of cancer cells (29). Trained immune cells display some similar metabolic characteristics to cancer cells, such as increased glycolysis, amino acid metabolism, and so on (27, 31). We speculated that proline metabolism may play an important role in the trained macrophages. As expected, proline displayed markedly difference among “up” metabolites in the volcano map (**Figure 4A**). The quantity of proline in PVM-OVA group was more than 3 times higher than that in PBS group (**Figure 4B**, P<0.001). In the proline synthesis pathway from glutamine as shown in **Figure 4C**, L-Glutamyl-P, a key intermediate metabolite, increased in PVM-OVA group, compared with that in PBS group (**Figure 4D**, P<0.05). In addition, some dipeptides containing glutamate or glutamine, such as Gamma−Glu−Leu, Gamma−Glutamylphenylalanine and Glutamylisoleucine also increased in PVM-OVA group, compared with those in PBS group (Fig 4D). These data imply that the proline synthesis pathway was up-regulated in PVM-OVA group. Glutaminase (GLS) is the first rate-limiting enzyme in the proline synthesis pathway, which catalyzes catabolism of glutamine. Proline - 5 - carboxylic acid reductase 1 (PYCR 1) catalyzes the last step of proline synthesis(**Figure 4C**). The relative expression of GLS1 or GLS2 significantly increased in PVM-OVA group, compared with that in PBS group, which was consistent with the change of proline (**Figure 4E**, P<0.05). However, the expression of PYCR1 was obviously reduced in PVM-OVA group, compared with that in PBS group (**Figure 4E**, P<0.05), which may be due to feedback inhibition of increased proline (32). All of the data suggested that proline synthesis pathway is remarkably active in PVM-OVA group.

### Proline metabolism plays a crucial role in development of the trained macrophages

**Figure 5:**
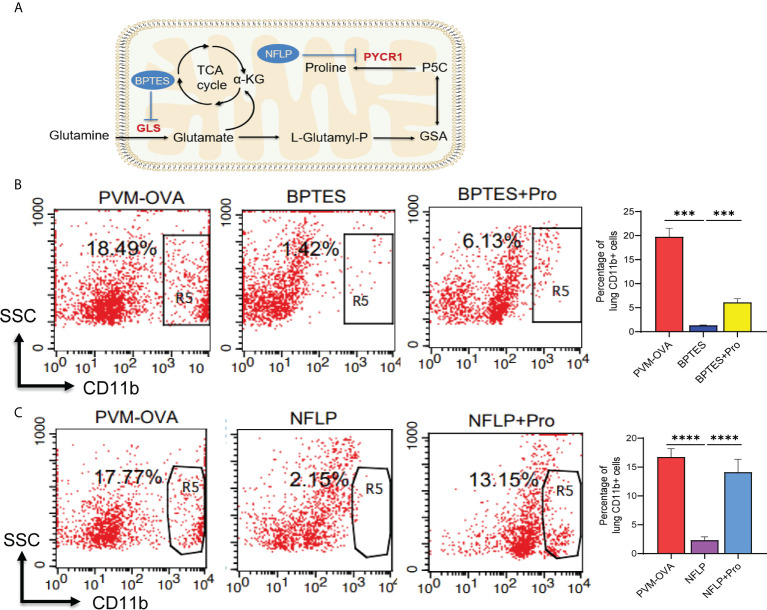
Proline metabolism plays a crucial role in development of the trained macrophages. **(A)** Diagram of proline synthesis from glutamine with inhibitors of key enzymes. **(B, C)** Mice were sensitized with PVM-OVA, PVM-OVA followed by BPTES or BPTES+Proline, or PVM-OVA followed by NFLP or NFLP+Proline. The lung CD11b+ trained macrophages in the memory phase were detected by flow cytometry (n = 5 per group). Each experimental group contained three biology repeats. ***P<0.001, ****P<0.001.

To explore the role of proline metabolism on development of the trained macrophages, mice were given BPTES or NFLP, a chemical inhibitor of GLS or PYCR1 in the synthetic pathway of proline (**Figure 5A**), or replenished proline after given BPTES or NFLP, during the early sensitization with PVM-OVA, and the trained macrophages were detected by flow cytometry. Compared with PVM-OVA group, the lung trained macrophages in BPTES group were significantly inhibited (Fig 5B, P<0.05). The percentage of trained macrophages in BPTES group was more than ten times lower than that in PVM-OVA group (**Figure 5B**, P<0.05). Expectedly, the trained macrophages in BPTES+Pro group was partly recovered (**Figure 5B**, P<0.05). Similarly, the lung trained macrophages in NFLP group were eight times less than those in PVM-OVA group, while those in NFLP +Pro group were almost fully recovered (**Figure 5C**, P<0.05). These results suggest that proline metabolism plays a crucial role in development of the trained macrophages.

### Excessive proline synthesis caused by early respiratory virus infection combined with allergen sensitization contributed to allergic asthma in childhood

**Figure 6:**
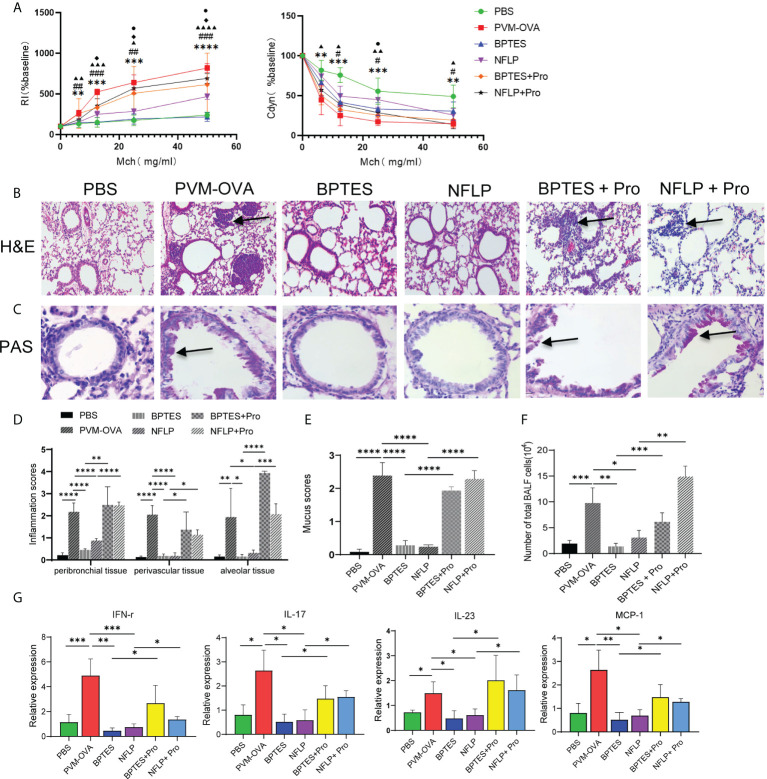
Excessive proline synthesis caused by early respiratory virus infection combined with allergen sensitization contributed to allergic asthma in childhood. Mice were sensitized with PVM-OVA, PVM-OVA followed by BPTES or NFLP, or PVM-OVA followed by BPTES+Proline or NFLP+Proline, and challenged with OVA after 21 days post infection. **(A)** RI and Cdyn to methacholine (MCh, 6.25, 12.5, 25, and 50 mg/ml), (#, ▲, ◆P < 0.05, * *,##, ▲▲P < 0.01, * * *,###, ▲▲▲P < 0.001. PVM-OVA group compared to PBS group, *, or BPTES group, #, or NFLP group, ▲. BPTES group compared to BPTES +Pro group, ◆. NFLP group compared to NFLP +Pro group, ●). **(B)** H&E staining shows peribronchiolar, perivascular, and interstitial inflammatory cell infiltration. (100 X). **(C)** PAS staining shows bronchiolar mucus production (400X). **(D, E)** Scores for pulmonary inflammation or mucus. **(F)** Number of inflammatory cells in BALF. **(G)** Relative expression of IFN-γ, IL-17, IL-23 and MCP-1 by real-time PCR. Each experimental group contained three biology repeats. Data are presented as means standard deviations of five mice per group and are representative of two experiments. *P<0.05, **P<0.01, ***P<0.001, ****P<0.0001. ▲▲▲▲ means PVM-OVA group compared with NFLP group (P<0.0001).

To study the effects of proline on the allergic asthma, BPTES or NFLP was injected into mice when they were sensitized by PVM-OVA in early life, and airway hyperresponsiveness and lung inflammation of the mice were evaluated following OVA challenge in childhood. As expected, inhibition of the key enzyme GLS or PYCR1 in the synthetic pathway of proline using inhibitor BPTES or NFLP when PVM-OVA sensitization in early life significantly inhibited RI, and increased Cdyn following OVA challenge in childhood, compared with PVM-OVA sensitization alone (**Figure 6A**, P<0.05). In contrast, RI increased in BPTES+Pro or NFLP+Pro group with supplement of proline, compared with that in BPTES or NFLP group at high doses of Mch, and Cdyn deduced in NFLP+Pro group at 25mg/mL Mch compared with that in NFLP group (**Figure 6A**, P<0.05). Compared with the severe inflammatory histopathology and mucus in PVM-OVA group, no or little inflammatory cell infiltration and mucus production were observed in BPTES or NFLP group, while the inflammatory cell and mucus almost recovered in BPTES+Pro or NFLP+Pro group (**Figures 6B-E**, P<0.05). Similarly, the total number of inflammatory cells in BALF remarkably reduced in BPTES or NFLP group compared with that in PVM-OVA group, while that in BPTES+Pro or NFLP+Pro recovered (**Figure 6F**, P<0.05). Then, the relative expression of Th1 type cytokine IFN-γ, Th17 type cytokine IL-17 and IL-23, and chemokine MCP-1 was detected by real-time RT-PCR. BPTES or NFLP significantly inhibited the expression of IFN-γ, IL-17, IL-23 and MCP-1, while proline supplementation could up-regulate the expression of these Th1/Th17 type and pro-inflammatory cytokines (**Figure 6G**, P<0.05). These results showed that inhibition of proline metabolic pathway could inhibit Th1/Th17 type responses, alleviate the symptoms of allergic asthma, while proline supplementation after the inhibition could restore the Th1/Th17 type response and asthmatic symptoms, which indicated that excessive proline synthesis caused by early respiratory virus infection combined with allergen sensitization contributed to allergic asthma in childhood. Regulation of proline metabolism may be a well preventive strategy for allergic asthma.

## Discussion

**Figure 7:**
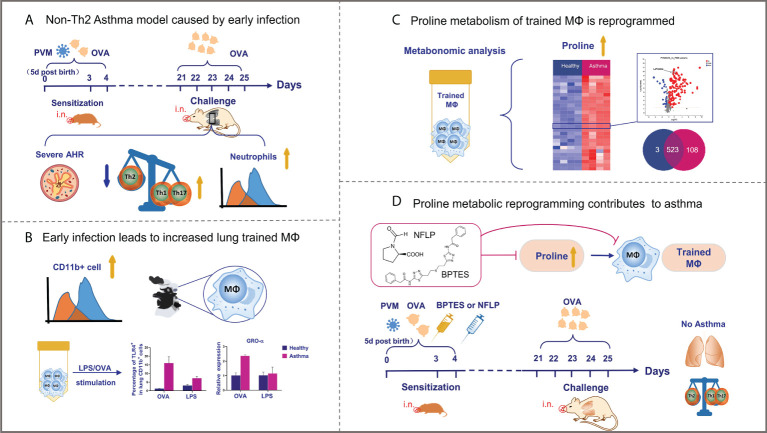
Graphical representation of results. **(A, B)** Neonatal respiratory virus infection can promote the occurrence of non-Th2 allergic asthma through the production of a large number of trained macrophages, which undergo metabolic reprogramming. **(C, D)** Notably, proline metabolic reprogramming mediate development of the trained macrophages as well as non-Th2 allergic asthma in childhood mice.

In this study, we established an animal model of neonatal respiratory virus infection promoting childhood non-Th2 (Th1/Th17) type allergic asthma. Neonatal sensitization with PVM-OVA resulted in increased lung trained macrophages, which mediated the childhood asthma. Interestingly, proline metabolism reprogramming was proved to play a critical role in development of the trained macrophages and allergic asthma. Inhibition of the excessive proline synthesis prevented allergic asthma in childhood (**Figure 7**). To our knowledge, we provide the first evidence that proline metabolism reprogramming contributes to trained macrophages caused by early respiratory virus infection and allergen sensitization, and the non-Th2 (Th1/Th17) type allergic asthma in childhood. Proline metabolism may be a target for preventing the allergic asthma in childhood.

Allergic asthma is a complex chronic airway disease in which many different immune cells and environmental exposure factors are involved in its pathological process (33). Development of childhood asthma is related to the lower respiratory tract viral infections in early years, and then exposed to allergens (34). Human RSV is the most frequent cause of respiratory tract infections in infants and are major triggers of wheezing and asthma exacerbations (35). In this study, PVM (a mouse equivalent strain of human RSV) infection combined with OVA exposure in early life led to severe allergic asthma following OVA challenge in juvenile mice, while PVM infection or OVA sensitization alone did not elicit classical asthma. Consistently, R S Peebles Jr et al. found that RSV infection during OVA sensitization increased and prolonged AHR compared to mice only RSV-infected or OVA-sensitized (22). Two asthma phenotypes have been described, Th2 and non-Th2. Th2-asthma (i.e., eosinophilic asthma) is characterized by elevated expression of Th2-type cytokines, along with airway eosinophilia, and augmented allergen-specific IgE. Non-Th2 asthma (i.e., neutrophilic asthma), by contrast, is characterized by high levels of IFN-γ and IL-17, along with airway neutrophilic infiltrate (36). Recent studies have increasingly shown that severe childhood asthma is associated with neutrophils (37, 38). In this study, the asthma mice showed increased Th1- and Th17-type responses and neutrophils in lungs, and no detectable IgE in sera, which is similar to the no-Th2 asthma. Consistently, RSV infection combined with OVA sensitization led to increased AHR with diminished Th2 response (22). In addition, human rhinovirus exposure combined with OVA sensitization led to Th1- and Th17-biased responses and neutrophilic inflammation following OVA challenge in mice (39). In contrast to Th2-asthma, non-Th2 asthma does not respond steroids or newly developed asthma drugs (40). Elucidating the underlying cellular and molecular mechanisms of the childhood asthma associated with early respiratory virus infection is critical for developing effective asthma treatments and predicting patient prognosis.

Immune memory plays a key role in the pathogenesis of allergic asthma. Immune memory is a defining characteristic of adaptive immunity, but in recent years, evidences have demonstrated that innate immune system can display characteristics of immunological memory, termed “innate immune memory” or “trained immunity” (12–14). Bovis bacillus Calmette-Guerin (BCG), Candida albicans, Candida albicans-derived beta-glucan, and oxidized low-density lipoprotein (oxLDL) have successfully induced innate immune training of macrophages (41–43). Trained monocytes/macrophages showed the increase in the production of pro-inflammatory cytokines following re-stimulation with LPS (44). Macrophages are the key cell group of immune tolerance and immune response in lungs (45) and have been considered to play an important role in the pathogenesis of virus-induced asthma exacerbations (46). In this study, the trained macrophages induced by PVM-OVA significantly increased, and showed enhanced ability to produce surface activated molecules and pro-inflammatory cytokines following LPS or OVA re-stimulation. Although being considered as a beneficial response against re-infections, trained immunity could also promote maladaptive immune responses that aggravate pathology (47). It has been reported that trained immune cells participate in many inflammatory diseases including asthma, atherosclerosis and neurodegenerative diseases (48, 49). We found that adoptive transfer of the trained macrophages induced by PVM-OVA resulted in asthma symptoms following OVA challenge. The results indicate that trained macrophages in lungs facilitate allergic asthma in childhood.

Trained immune cells are mainly characterized by metabolic and epigenetics reprogramming (12). Metabolic reprogramming, such as glycolysis, glutaminolysis, or cholesterol synthesis, is critical for the functional fate of trained monocytes and macrophages (50). Beta-glucan triggers metabolic reprogramming in macrophages, and induces the expression of genes associated with central metabolism, enabling innate immune cells to mount a highly efficient proinflammatory response against recurrent fungal infection (51). We found that the trained macrophages in PVM-OVA group had a large number of metabolites, such as amino acids, phospholipids, isocitrate, and so on. Amino acids play a vital role in the physiological/metabolic functions of humans, and the abnormal metabolism of amino acids is related to many human diseases, including asthma (52) and cancer (53). The increase of certain amino acids such as cysteine and serine in asthmatic patients leads directly or indirectly to the onset of asthma (54). The metabolic profiles of amino acids in asthmatic children were significantly different from those of normal children (55). Trained immune cells have many similar metabolic characteristics to cancer cells, for example, they are both energy-intensive cells, and have metabolic disorders, including amino acid and glucose metabolism disorders (56–58). The disorder of proline biosynthesis promotes the occurrence of various cancers, and proline is the “limiting amino acid” of cancer cells (29, 59). In this study, the level of proline in the trained macrophages induced by PVM-OVA was significantly increased, compared with that in naïve macrophages. Moreover, some intermediates as well as the key enzyme GLS in the biosynthesis pathway of proline were also significantly increased in the trained macrophages. The last enzyme PYCR1 in the biosynthesis pathway of proline reduced in the trained macrophages, which is consistent with the previous report that PYCR1 activity was significantly feedback inhibited by increased proline (32). Inhibition of the key enzyme GLS or PYCR suppressed induction of the trained macrophages, and led to completely prevention of allergic asthma in childhood. In contrast, supplement of proline after delivery of the inhibitors led to remarkable restore of the trained macrophages, as well as allergic asthma. These results indicated that proline metabolism plays a crucial role in development of the trained macrophages as well as childhood allergic asthma. Proline may be a “ limiting amino acid” for trained immune cells. However, this study did not firmly establish the causative effects of proline metabolism in trained macrophages in promoting later asthma development, which is a limitation of this study. In sum, to our knowledge, this is the first report that proline metabolic reprogramming is involved in the training of macrophages in lungs associated with early respiratory virus infection combined with allergen sensitization, as well as the development of the non-Th2 (Th1/Th17) type allergic asthma in childhood. Inhibition of excessive proline synthesis prevented the childhood allergic asthma associated with early respiratory infection combined with allergen sensitization. Proline metabolism could be a well target for preventing allergic asthma in childhood and predicting patient prognosis.

## Data availability statement

The raw data supporting the conclusions of this article will be made available by the authors, without undue reservation.

## Ethics statement

The animal study was reviewed and approved by Laboratory Animal Ethical and Welfare Committee Hebei Medical University, Hebei Medical University.

## Author contributions

HL performed, analyzed experiments and wrote the manuscript. LM, WL and BZ performed, analyzed experiments. JW, SC, YW, FG and BQ took part in experiments. XZ and YD discussed data. RZ designed experiments, analyzed experiments, wrote the manuscript, and provided overall direction. All authors contributed to the article and approved the submitted version.

## Funding

This work was supported by grants from National Natural Science Foundation of China (NO. 81671635); Natural Science Foundation of Hebei province (NO. H2020206510 and H2016206473).

## Conflict of interest

The authors declare that the research was conducted in the absence of any commercial or financial relationships that could be construed as a potential conflict of interest.

## Publisher’s note

All claims expressed in this article are solely those of the authors and do not necessarily represent those of their affiliated organizations, or those of the publisher, the editors and the reviewers. Any product that may be evaluated in this article, or claim that may be made by its manufacturer, is not guaranteed or endorsed by the publisher.
